# *Wolbachia* endosymbionts in *Drosophila* regulate the resistance to Zika virus infection in a sex dependent manner

**DOI:** 10.3389/fmicb.2024.1380647

**Published:** 2024-06-05

**Authors:** Ghada Tafesh-Edwards, Margarita Kyza Karavioti, Klea Markollari, Dean Bunnell, Stanislava Chtarbanova, Ioannis Eleftherianos

**Affiliations:** ^1^Infection and Innate Immunity Laboratory, Department of Biological Sciences, The George Washington University, Washington, DC, United States; ^2^Department of Biological Sciences, The University of Alabama, Tuscaloosa, AL, United States

**Keywords:** Zika virus, *Drosophila melanogaster*, *Wolbachia*, innate immunity, infection, immune signaling

## Abstract

*Drosophila melanogaster* has been used extensively for dissecting the genetic and functional bases of host innate antiviral immunity and virus-induced pathology. Previous studies have shown that the presence of *Wolbachia* endosymbionts in *D. melanogaster* confers resistance to infection by certain viral pathogens. Zika virus is an important vector-borne pathogen that has recently expanded its range due to the wide geographical distribution of the mosquito vector. Here, we describe the effect of *Wolbachia* on the immune response of *D. melanogaster* adult flies following Zika virus infection. First, we show that the presence of *Wolbachia* endosymbionts promotes the longevity of uninfected *D. melanogaster* wild type adults and increases the survival response of flies following Zika virus injection. We find that the latter effect is more pronounced in females rather than in males. Then, we show that the presence of *Wolbachia* regulates Zika virus replication during Zika virus infection of female flies. In addition, we demonstrate that the antimicrobial peptide-encoding gene *Drosocin* and the sole Jun N-terminal kinase-specific MAPK phosphatase *Puckered* are upregulated in female adult flies, whereas the immune and stress response gene *TotM* is upregulated in male individuals. Finally, we find that the activity of RNA interference and Toll signaling remain unaffected in Zika virus-infected female and male adults containing *Wolbachia* compared to flies lacking the endosymbionts. Our results reveal that *Wolbachia* endosymbionts in *D. melanogaster* affect innate immune signaling activity in a sex-specific manner, which in turn influences host resistance to Zika virus infection. This information contributes to a better understanding of the complex interrelationship between insects, their endosymbiotic bacteria, and viral infection. Interpreting these processes will help us design more effective approaches for controlling insect vectors of infectious disease.

## Introduction

Symbiotic interactions between microbes and animals are common in nature. The role of symbionts in providing nutrients missing in the diets of various animals has been known for many years. Such symbionts are particularly common in insects, perhaps because most insects are specialist herbivores and plants are frequently poor-quality food for animals; the essential elements that the animals lack can be provided by symbiotic microbes. In many cases, the association has become so close that the microbial partner (usually a bacterium) lives within cells in the host’s body (it is then said to be an endosymbiont), is maternally transmitted from one host generation to another, and is never found in the free-living condition (Dale and Moran, 2006). Although such relationships have been most commonly studied with respect to nutritional effects on the host, there can be other benefits. For example, endosymbiotic bacteria may protect their hosts from parasites or pathogens (Brownlie and Johnson, 2009; Eleftherianos et al., 2013). The most widespread and widely studied endosymbionts are *Wolbachia*, which are harbored by more than half of all insect species and are able to manipulate the reproductive properties of their hosts, while in other hosts such as bedbugs or nematodes they can act as nutritional mutualists (Pietri et al., 2016; Landmann, 2019; Bi and Wang, 2020; Newton and Rice, 2020).

*Wolbachia* are Gram-negative obligate intracellular Alphaproteobacteria bacteria that are found in the germline and somatic tissues of most arthropod species, and they are transmitted maternally from infected mothers (Porter and Sullivan, 2023). Also, they establish an endosymbiotic relationship with several insect species, including *D. melanogaster* (Kaur et al., 2021). In fruit flies, *Wolbachia pipientis* endosymbionts modulate diverse biological processes, such as reproduction, nutrition, development, and longevity, offering many benefits to the host (Iturbe-Ormaetxe and O’Neill, 2007). Research on *Wolbachia* has important implications for understanding the molecular and functional bases of bacterial symbiosis, and also produces significant information on the regulation of host–microbe interactions which can be potentially used in translational applications in agriculture and biomedicine (Yen and Failloux, 2020; Edenborough et al., 2021; Araújo et al., 2022).

One of the crucial roles of *Wolbachia* in insects is the immune protection against certain viral pathogens known as a pathogen-blocking effect, which is probably attributed to the activation of host immunity or competition with virus for cellular resources (Pimentel et al., 2021). It was originally demonstrated that the presence of *Wolbachia* in *D. melanogaster* increases resistance to infection by three insect RNA viruses (Drosophila C virus, Nora virus and Flock House virus) but not to infection by a DNA virus (Insect Iridescent Virus 6) (Hedges et al., 2008; Teixeira et al., 2008). *Wolbachia*-mediated antiviral protection in *Drosophila* species has been demonstrated for a number of different *Wolbachia* strains (Martinez et al., 2014, 2017). For example, when dengue viruses are injected into *D. melanogaster*, virus accumulation is significantly reduced in the presence of the non-virulent *Wolbachia* strain wMel and the life-shortening strain wMelPop-CLA (Rancès et al., 2012). Interestingly, Toll and Immune deficiency (Imd) pathways are not required for expression of the dengue virus-blocking phenotype in the *Drosophila* host and *Wolbachia* endosymbionts do not interact with the Toll pathway-mediated resistance to viral oral infection (Rancès et al., 2013; Ferreira et al., 2014). However, *Wolbachia* decreases the biodiversity of the gut microbiota without changing the total microbial load, and altering the gut microbiota composition with antibiotic treatment increases *Wolbachia* density without boosting the resistance against *Drosophila* C Virus (DCV) (Simhadri et al., 2017; Ye et al., 2017). In terms of systemic viral infection, extracellular signal-regulated kinases (ERK) signaling activity increases in the presence of *Wolbachia* without affecting the protection of the host to systemic infection with DCV (Wong et al., 2016). When *D. melanogaster* adults are maintained on cholesterol-enriched diets and they contain the *Wolbachia* strains wMelPop and wMelCS, the flies exhibit reduced pathogen blocking and higher viral copy number compared to flies grown on standard diet (Caragata et al., 2013). Subsequent studies further revealed that stable transinfection of *D. melanogaster* with highly protective *Wolbachia* strains is not necessarily associated with general immune activation (Chrostek et al., 2014). Although DCV infection causes increased sleep in *D. melanogaster* females than in males flies, the presence of *Wolbachia* does not affect this behavioral response (Vale and Jardine, 2015). *Wolbachia* can suppress the evolution of *D. melanogaster* resistance genes because in the presence of the endosymbiotic bacteria, the resistant allele of *pastrel-a* gene, which has a major effect on resistance to DCV, is at a lower frequency than in the symbiont-free individuals (Martinez et al., 2016).

Zika virus is a vector-borne flavivirus that has become a significant threat to human health. The disease was originally limited to African countries, however, Zika virus cases have been reported in other parts of the world, such as Brazil and Malaysia (Plourde and Bloch, 2016). The *Flaviviridae* family comprises several notable viruses, including dengue virus and yellow fever virus, all of which are transmitted primarily through mosquito vectors and can cause significant public health concerns (Gubler et al., 2017). The connection between *D. melanogaster* and Zika virus lies in the use of fruit flies as a model organism to study Zika virus pathogenesis and the host innate immune response against flaviviruses. By introducing Zika virus into adult *D. melanogaster*, it is possible to identify important genes and pathways involved in the host response to viral infection, providing valuable information on host-virus interactions and the underlying antiviral mechanisms (Tafesh-Edwards and Eleftherianos, 2020a). For example, a recent study in which *Drosophila* flies were subjected to injection with Zika virus has demonstrated activation of the Imd pathway in the brain (Liu et al., 2018). More precisely, *Diptericin* (read-out of Imd signaling), but not *Drosomycin* (read-out of Toll signaling), is upregulated in the heads of Zika virus-infected flies, and this result is not observed in fly null mutants for the transcription factor Relish. In addition, the *Drosophila* Imd pathway in the fly brain appears to be required to restrict Zika virus infection in this tissue (Lye and Chtarbanova, 2018). Zika virus infection induces antiviral autophagy in the brain, a process that is Relish-dependent (Liu et al., 2018). The fly ortholog of the mammalian polyubiquitin-binding scaffold protein p62, the autophagy cargo receptor Ref(2)P, which is a known restriction factor for natural viral pathogens of the fly such as the *Drosophila* Sigma virus (Contamine et al., 1989; Longdon et al., 2010), is also directed against Zika virus in the brain, because its knockdown increases the rate of Zika virus replication in fly heads (Liu et al., 2018), and protection against Zika virus is not dependent on RNAi in the fly brain (Liu et al., 2018). However, we have recently shown that *Dicer2* loss-of-function mutant flies have increased sensitivity to Zika virus injection into the thorax and exhibit higher viral loads (Harsh et al., 2018).

Activation of the Imd pathway triggers signaling through the adaptor IMD protein and various caspases and kinases, resulting in the induction of c-Jun N-terminal kinase (Jnk) signaling, which forms one of the two functional branches of the Imd pathway (Sluss et al., 1996; Chen et al., 2002). Although the role of Jnk signaling in the regulation of the immune response against bacterial pathogens through modulating the expression of antimicrobial peptides and maintaining host homeostasis is well documented (Tafesh-Edwards and Eleftherianos, 2020b), its participation in the immune response of the fly against viral infections is lagging. Intriguingly, the Jnk pathway has a broad antiviral function against dengue, Zika, and chikungunya viruses, which is mediated by the complement system and apoptosis in the *Aedes aegypti* salivary glands (Chowdhury et al., 2020).

The contribution of Janus kinase–signal transducer and activator of transcription (Jak/Stat) signaling to the *D. melanogaster* antiviral immune response is virus specific (Tafesh-Edwards and Eleftherianos, 2020a). Although the Jak/Stat pathway can be induced by RNA viruses, such as DCV, Cricket Paralysis Virus (CrPV), Flock House Virus (FHV), and *Drosophila* X Virus (DXV), it is only required for resistance against the two Dicistroviruses, DCV and CrPV (Dostert et al., 2005; Myllymäki and Rämet, 2014; Chow and Kagan, 2018; Huang et al., 2023). Also, we have previously documented that Zika virus infection induces negative regulation of Jak/Stat signaling, and Zika virus non-structural protein 4A (NS4A) interacts with Jak/Stat signaling components (Harsh et al., 2020).

Here we explored the role of *Wolbachia* endosymbionts in the *D. melanogaster* immune response against Zika virus infection. For this, we tested the survival ability of wild type adult flies carrying *Wolbachia* endosymbionts during Zika virus infection and the viral replication in these individuals. We also assessed whether the presence of *Wolbachia* alters the innate immune signaling activity upon challenge with Zika virus. First, we find a positive correlation between the survival ability of female and male *D. melanogaster* following Zika virus infection and the presence of *Wolbachia* endosymbionts. Then, we show that females lacking *Wolbachia* have a higher Zika viral load than females carrying *Wolbachia*, which implies the participation of *Wolbachia* in resistance to Zika virus infection. Effects on fly survival and Zika virus load could be attributed to changes in the regulation of innate immune signaling, as females containing *Wolbachia* have increased Imd pathway activity and males containing *Wolbachia* have increased Jak/Stat pathway activity. This information is vital because it provides valuable insights into the interconnection between bacterial endosymbiosis in insects, viral pathogenesis, and host defense mechanisms. Similar research can contribute toward development of approaches for the management of vectors of infectious diseases.

## Materials and methods

### Fly stocks

Two natural Canton-S lines of *D. melanogaster* containing or lacking *Wolbachia pipientis* (strain *w*Mel) were used in all experiments. Flies were reared on ready-made fly food (LabExpress, Ann Arbor, MI, United States) supplemented with yeast (Carolina Biological Supply, Burlington, NC, United States). All vials were maintained in an incubator at 25°C and 12 h light/12 h dark photoperiod cycle. Both *D. melanogaster* lines were amplified for experimentation by transferring adult flies to fresh vials every third day. Female and male flies were selected from the same generation and randomly assigned to experimental groups.

### Zika virus stocks

Stocks of Zika virus strain MR766 were maintained and amplified as described before (Harsh et al., 2018).

### Fly longevity experiments

Ten male and 10 female Canton-S newly eclosed *D. melanogaster* adult flies carrying or lacking *Wolbachia* endosymbionts, were kept in separate vials containing fly food at 25°C and a 12:12-h light:dark photoperiod cycle. Observations were held at 24-h intervals to record fly survival over a 100-day period. All flies were transferred to fresh vials every 3 days. The experiment was repeated five times with three replicates for each experimental treatment. In total, 150 female and 150 male flies were used in the longevity experiments.

### Fly survival experiments

Ten female and 10 male 3–5 days old flies of the two Canton-S lines carrying or lacking *Wolbachia* endosymbionts were first anesthetized with carbon dioxide for a few seconds and then injected into the thorax with 100 nL of a Zika virus solution (11,000 PFU/fly) in PBS (pH 7.5) using a Nanojector apparatus (Nanoject III Programmable Nanoliter Injector, Drummond Scientific, Broomall, PA, United States). Flies injected with 100 nL of PBS served as negative controls. Virus-infected and control flies were maintained in vials with fly food at 25°C and they were transferred to fresh vials every third day for the duration of the survival experiment. Survival results were estimated at 24-h intervals for up to 25 days. Three independent experiments were conducted, and each experiment included three replicates for each experimental condition.

### Gene expression analysis

Ten female and 10 male Canton-S 3–5 days old flies containing or lacking *Wolbachia* were injected with either Zika virus or PBS only (negative control), as described above, and at 4 days post-injection were collected and stored at −80°C. A pool of 10 flies was homogenized using plastic pestles and RNA isolation was carried out using TRIzol (Invitrogen, Waltham, MA, United States) according to the manufacturer’s protocol, followed by complementary DNA (cDNA) synthesis using the AB High-Capacity cDNA Reverse Transcription Kit (Fisher Scientific, Hampton, NH). Zika virus load and immune gene expression were estimated using Zika virus gene-specific primers and *D. melanogaster* gene-specific primers, respectively (Table 1). Gene expression analysis was assessed using quantitative Real Time PCR (qRT-PCR) and two technical replicates per treatment on a CFX96 Real-Time PCR detection system (Bio-Rad Laboratories, Hercules, CA). Cycle conditions were 95°C for 2 min, 40 repetitions of 95°C for 15 s, 61°C for 30 s, 95°C for 15 s, 65°C for 5 s, and 95°C for 5 s. Quantitative RT-PCR experiments were repeated three times with each experiment consisting of three biological replicates and two technical replicates per condition. In total, 360 female and 360 male flies were used in the gene expression analysis experiments.

**Table 1:** Primer sequences used for quantitative PCR.

Gene	Accession number	Pathway	Forward primer (5′ to 3′)	Reverse primer (3′ to 5′)
*Ago-2*	CG7439	RNAi	CCGGAAGTGACTGTGACAGATCG	CCTCCACGCACTGCATTGCTCG
*Basket*	CG5680	Jnk	GACAGCTCAGCACCAACACT	GCTTGGCATGGGTTACATTT
*Defensin*	CG1385	Toll	CGCATAGAAGCGAGCCACATG	CGCATAGAAGCGAGCCACATG
*Drosocin*	CG10816	Imd	TTCACCATCGTTTTCCTGCT	AGCTTGAGCCAGGTGATCCT
*Dicer*-2	CG6493	RNAi	GTATGGCGATAGTGTGACTGCGAC	GCAGCTTGTTCCGCAGCAATATAGC
*Diptericin*	CG10794	Imd	TGCGCAATCGCTTCTAC	GTGGAGTGGGCTTCATG
*Drosomycin*	CG10810	Toll	TGAGAACCTTTTCCAATATGATG	CCAGGACCACCAGCAT
*Puckered*	CG7850	Jnk	GGCCTACAAGCTGGTGAAAG	AGTTCAGATTGGGCGAGATG
*TotA*	CG31509	Jak/Stat	GAAGATCGTGAGGCTGACAAC	GTCCTGGGCGTTTTTGATAA
*TotM*	CG14027	Jak/Stat	GCTGGGAAAGGTAAATGCTG	AGGCGCTGTTTTTCTGTGAC
*RpL32*	CG7939	–	GATGACCATCCGCCCAGCA	CGGACCGACAGCTGCTTGGC
*ZIKV NS5*	055839	–	CCTTGGATTCTTGAACGAGGA	AGAGCTTCATTCTCCAGATCAA

### Statistical analysis

All data were processed using the GraphPad Prism5 software. Results from the longevity and survival experiments were statistically analyzed using a log-rank (Mantel-Cox) test and a chi-square test for pairwise comparison between each experimental group and the control group. Results from the gene expression analyses were conducted using a one-way analysis of variance (ANOVA) as well as a Tukey *post-hoc* test. Fold changes were estimated using the 2^-ΔΔC^_T_ method and *Ribosomal protein L32* (*RpL32*) as the housekeeping gene (Livak and Schmittgen, 2001; Schmittgen and Livak, 2008). PBS control treatments were used as a baseline for comparison in differential expression analysis. *p*-values less than 0.05 were considered significantly different. All error bars represent standard error of mean and all figures were generated using the GraphPad Prism5 software.

## Results

### Presence of *Wolbachia* prolongs the lifespan of *Drosophila melanogaster* Canton-S flies

**Figure 1:**
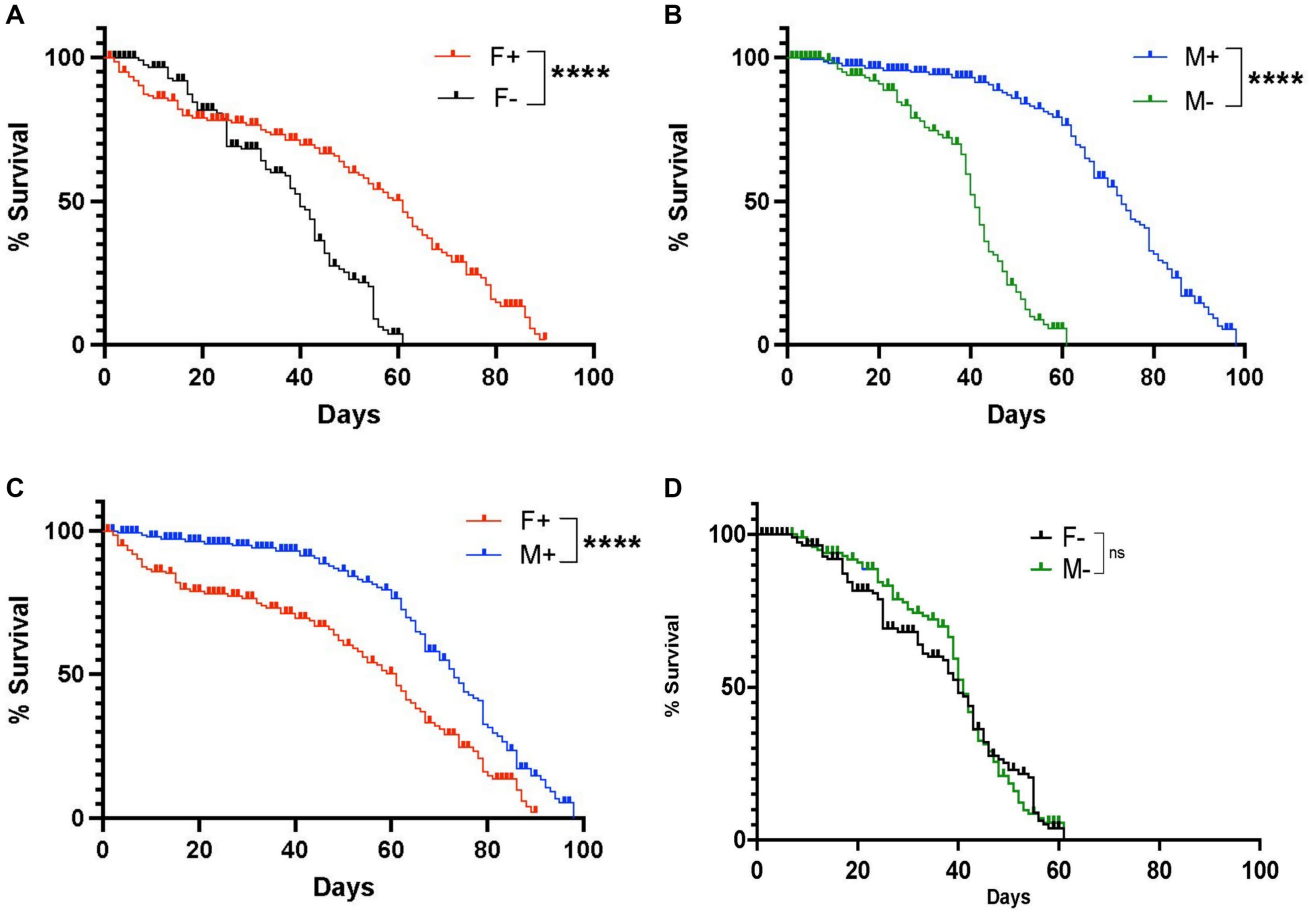
Lifespan of *Drosophila melanogaster* wild type adult flies containing or lacking *Wolbachia* endosymbionts. **(A)** Percent survival of *D. melanogaster* Canton-S female adult flies carrying (F+) or lacking (F−) *Wolbachia* endosymbionts (****p < 0.0001). **(B)** Percent survival of *D. melanogaster* Canton-S male adult flies carrying (M+) or lacking (M-) *Wolbachia* endosymbionts. **(C)** Percent survival of *D. melanogaster* Canton-S female and male adult flies carrying *Wolbachia* endosymbionts (F+ and M+, respectively) (****p < 0.0001). **(D)** Percent survival of *D. melanogaster* Canton-S female and male adult flies lacking *Wolbachia* endosymbionts (F− and M−, respectively); ns, non-significant difference (*n* = 150 flies of each sex).

We first assessed changes in longevity between *D. melanogaster* Canton-S male and female adult flies carrying or lacking *Wolbachia* endosymbionts. We found that female flies containing *Wolbachia* had a maximum lifespan of 91 days, whereas female individuals lacking *Wolbachia* had a maximum lifespan of 61 days, indicating a significant difference in longevity depending on the presence of the endosymbionts (Figure 1A). We also found that males containing *Wolbachia* had a maximum lifespan of 98 days, which was significantly longer compared to males without *Wolbachia* whose maximum lifespan was 61 days (Figure 1B). We further observed a significant difference in lifespan between males and females carrying *Wolbachia,* indicating a difference in longevity between the two sexes in the presence of the endosymbiont (Figure 1C), but not in its absence (Figure 1D). These results indicate that the presence of *Wolbachia* endosymbionts extends the longevity of *D. melanogaster* female and male wild type adult flies.

### *Drosophila melanogaster* Canton-S females carrying *Wolbachia* have increased survival response to Zika virus infection

**Figure 2:**
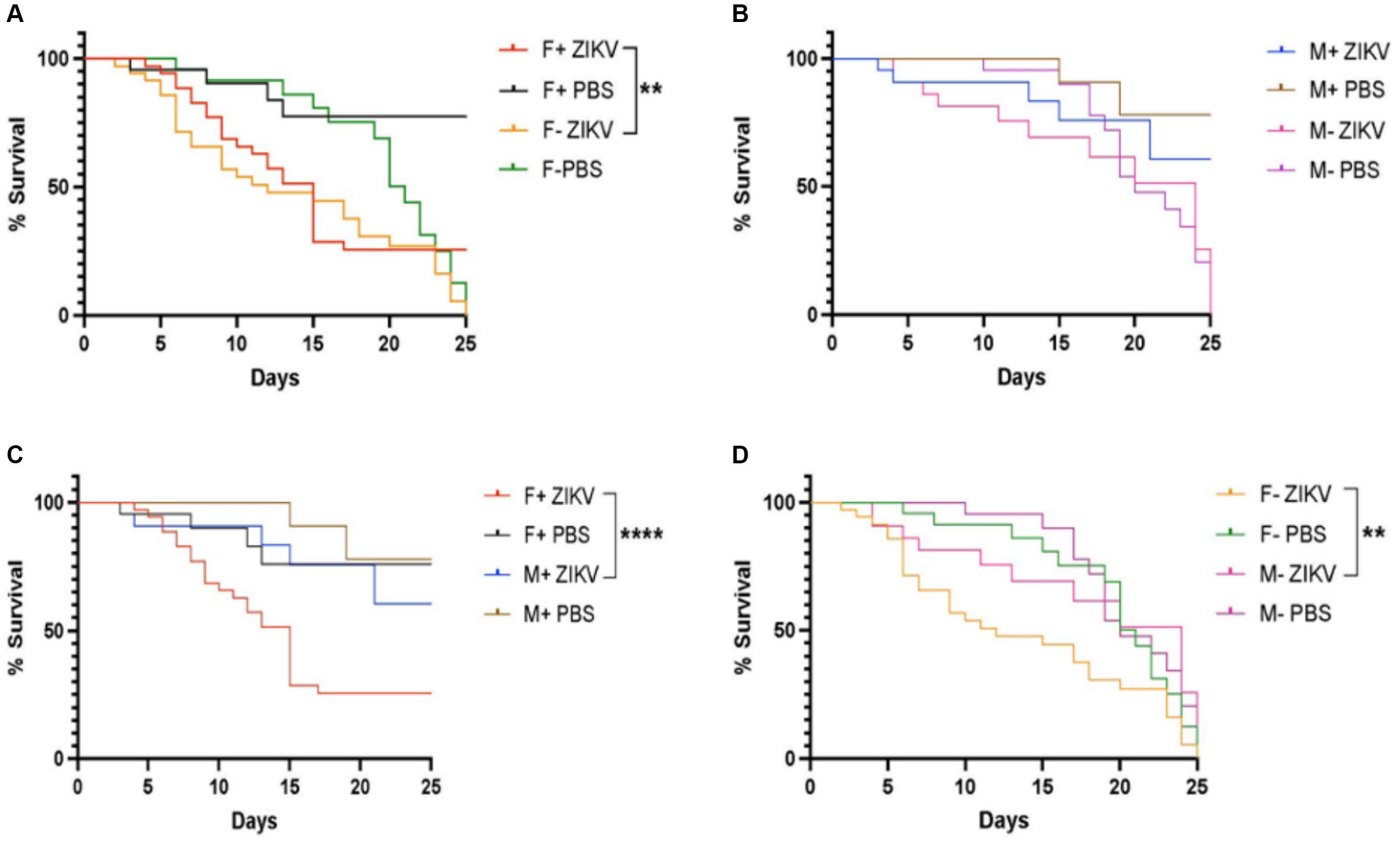
*Wolbachia* endosymbionts promote the survival of wild type *Drosophila melanogaster* against Zika virus infection. **(A)** Percent survival of *D. melanogaster* Canton-S female adult flies carrying or lacking *Wolbachia* endosymbionts following infection with Zika virus (ZIKV) (F+ ZIKV and F− ZIKV, respectively). Uninfected flies were injected with sterile PBS (F+ PBS and F− PBS) and acted as controls (**p < 0.01). **(B)** Percent survival of male flies carrying or lacking *Wolbachia* endosymbionts following infection with Zika virus (M+ ZIKV and M− ZIKV, respectively). Fly infections with PBS served as uninfected controls (M+ PBS and M− PBS). **(C)** Percent survival of *D. melanogaster* Canton-S female and male adult flies containing *Wolbachia* endosymbionts after infection with Zika virus (F+ ZIKV and M+ ZIKV, respectively). Uninfected control flies were injected with sterile PBS only (F+ PBS and M+ PBS) (****p < 0.0001). **(D)** Percent survival of *D. melanogaster* Canton-S female and male adult flies lacking *Wolbachia* endosymbionts following Zika virus infection (F− ZIKV and M− ZIKV, respectively). Uninfected flies were injected with sterile PBS (F− PBS and M− PBS) (**p < 0.01).

Next, we investigated the survival ability of *D. melanogaster* carrying and lacking *Wolbachia* endosymbionts following Zika virus infection through intrathoracic injection. We found that the presence of *Wolbachia* had a protective effect on female flies and the difference in survival compared to females lacking *Wolbachia* was significant (Figure 2A). However, we observed that males without *Wolbachia* were more sensitive compared to males carrying the endosymbionts, but the difference was not statistically significant (Figure 2B). We also found that males containing *Wolbachia* exhibited a significant better survival rate compared to their female counterparts (Figure 2C). Finally, there was a statistically significant difference in survival between males lacking *Wolbachia* and their corresponding female individuals (Figure 2D). Together, these results suggest that *Wolbachia* endosymbionts promote the survival of *D. melanogaster* adult female flies upon infection with Zika virus.

### Presence of *Wolbachia* in *Drosophila melanogaster* Canton-S females regulates Zika virus replication

**Figure 3:**
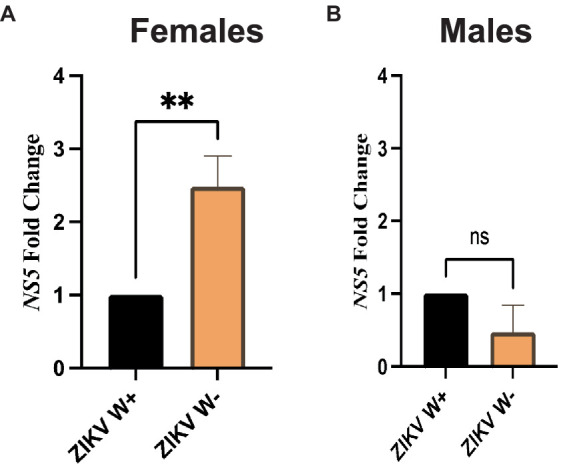
Zika virus replication in *Drosophila melanogaster* adults containing or lacking *Wolbachia* endosymbionts. **(A)** Expression of Zika virus (ZIKV) *NS5* in *D. melanogaster* Canton-S female adult flies carrying (W+) or lacking (W−) *Wolbachia* endosymbionts at 4 days following intrathoracic injection (**p < 0.01). **(B)** Expression of Zika virus (ZIKV) NS5 in *D. melanogaster* Canton-S male adult flies carrying (W+) or lacking (W−) *Wolbachia* endosymbionts at 4 days following virus infection (ns, non-significant difference; *n* = 360 flies of each sex). All data were normalized to the housekeeping gene *RpL32*, shown relative to flies injected with PBS.

We then examined whether the presence of *Wolbachia* in *D. melanogaster* female and male adult flies affects Zika virus load. For this, we used gene-specific primers to estimate the expression of Zika virus *NS5* methyltransferase, which encodes both the viral methyltransferase and RNA-dependent RNA polymerase (Elshahawi et al., 2019; Song et al., 2021). We found that female flies carrying *Wolbachia* had significantly lower expression of Zika virus *NS5* compared to females without the endosymbionts (Figure 3A). In contrast, there were no significant differences in Zika virus *NS5* fold change between males with *Wolbachia* and those lacking the endosymbiotic bacteria (Figure 3B). These results demonstrate that the effect of *Wolbachia* endosymbionts on Zika virus replication in *D. melanogaster* Canton-S is sex-specific and the presence of the endosymbionts confers resistance to female, but not male, adult flies.

### Presence of *Wolbachia* in *Drosophila melanogaster* Canton-S adult flies infected with Zika virus does not affect the expression of genes in the RNA interference pathway

**Figure 4:**
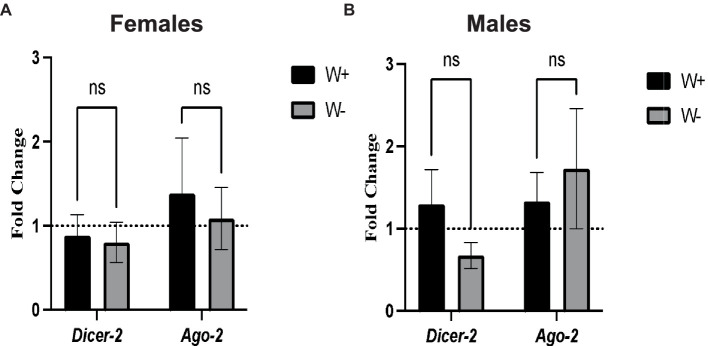
Expression of RNA interference pathway genes in Zika virus infected *Drosophila melanogaster* in the presence or absence of *Wolbachia* endosymbionts. **(A)** Expression of *Dicer-2* and *Ago-2* in *D. melanogaster* Canton-S female flies containing (W+) or lacking (W−) *Wolbachia* endosymbionts after Zika virus infection (ns, non-significant difference). **(B)** Expression of *Dicer-2* and *Ago-2* in *D. melanogaster* Canton-S male flies containing (W+) or lacking (W−) *Wolbachia* endosymbionts (ns, non-significant difference). Gene expression levels were normalized to the housekeeping gene *RpL32*. The horizontal dotted line indicates gene expression in uninfected controls treated with PBS (*n* = 360 flies of each sex).

We first tested whether the presence of *Wolbachia* in *D. melanogaster* adults infected with Zika virus modifies the transcriptional gene expression levels of *Dicer-2* and *Ago-2*. We found no significant changes in the transcript levels of *Dicer-2* and *Ago-2* between Zika virus infected female flies (Figure 4A) and male flies (Figure 4B) carrying or lacking *Wolbachia* endosymbionts. These results suggest that *Wolbachia* endosymbionts do not affect RNAi signaling activity in *D. melanogaster* adults in the context of Zika virus infection.

### Presence of *Wolbachia* in *Drosophila melanogaster* Canton-S infected with Zika virus upregulates the expression of *Drosocin*

**Figure 5:**
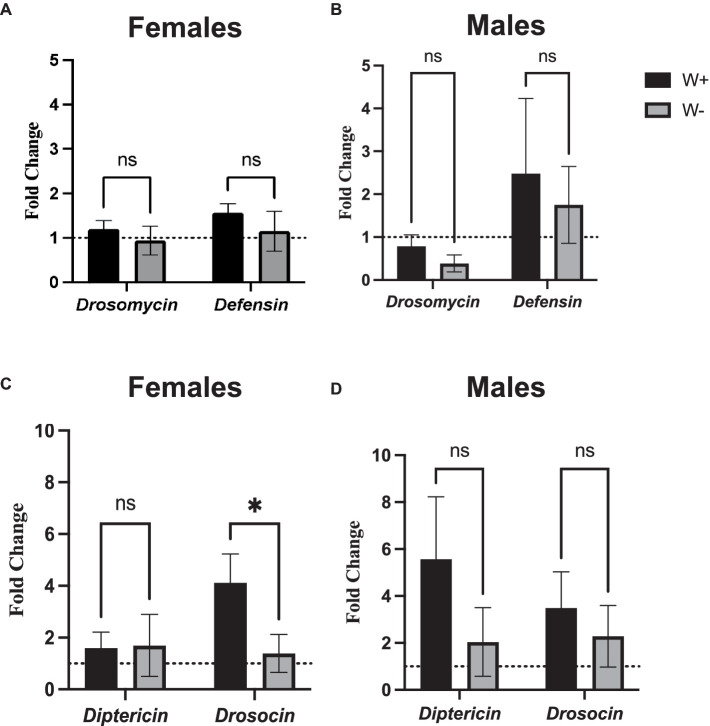
Toll and Immune deficiency pathway gene expression in Zika virus infected *Drosophila melanogaster* in the presence or absence of *Wolbachia* endosymbionts. Expression of *Drosomycin* and *Defensin* (Toll pathway) in *D. melanogaster* Canton-S **(A)** female and **(B)** male flies carrying (W+) or lacking (W−) *Wolbachia* endosymbionts following Zika virus infection (ns, non-significant differences). Expression of *Diptericin* and *Drosocin* (Imd pathway) in *D. melanogaster* Canton-S **(C)** female and **(D)** male flies carrying (W+) or lacking (W−) *Wolbachia* endosymbionts following Zika virus infection (*p < 0.01). Gene expression levels were normalized to the housekeeping gene *RpL32*. The horizontal dotted line indicates gene expression in uninfected controls treated with PBS (*n* = 360 flies of each sex).

To test whether the presence of *Wolbachia* affects the NF-κB signaling activity in *D. melanogaster* adults following intrathoracic challenge with Zika virus, we used qPCR and gene-specific primers to estimate the expression of frequently-measured readout genes in the Toll and Imd pathways. We observed no statistical differences in the expression levels of *Drosomycin* and *Defensin* (Toll pathway) between female or male flies carrying or lacking *Wolbachia* endosymbionts (Figures 5A,B). Interestingly, we noticed significantly higher levels of *Drosocin* expression, an antimicrobial peptide encoding gene that acts as readout for the regulation of Imd pathway, in female flies carrying *Wolbachia* compared to those lacking the endosymbionts (Figure 5C). The difference in *Drosocin* expression was not observed between male flies carrying or lacking the endosymbiotic bacteria (Figure 5D). Similarly, no changes in *Diptericin* expression were noted in female and male flies regardless of the presence or absence of *Wolbachia* (Figures 5C,D). These results imply that *Wolbachia* endosymbionts can induce the expression of certain antimicrobial peptide-encoding genes in Zika virus infected *D. melanogaster* adults in a sex-specific manner.

### Presence of *Wolbachia* in *Drosophila melanogaster* Canton-S adults infected with Zika virus alters Jak/Stat and Jnk signaling activity in sex-specific manner

**Figure 6:**
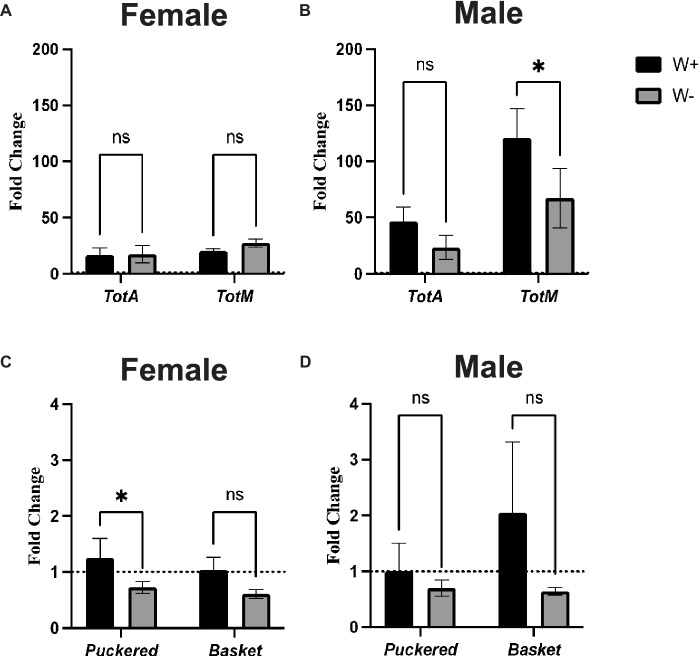
Jak/Stat and Jnk pathway activity in Zika virus infected *Drosophila melanogaster* in the presence or absence of *Wolbachia* endosymbionts. Expression of *TotaA* and *TotM* (Jak/Stat pathway) in *D. melanogaster* Canton-S **(A)** female and **(B)** male adult flies carrying (W+) or lacking (W−) *Wolbachia* endosymbionts following Zika virus infection (ns, non-significant differences). Expression of *Puckered* and *Basket* (Jnk pathway) in *D. melanogaster* Canton-S **(C)** female and **(D)** male flies carrying (W+) or lacking (W−) *Wolbachia* endosymbionts following Zika virus infection (*p < 0.01). Gene expression levels were normalized to the housekeeping gene *RpL32*. The horizontal dotted line indicates gene expression in uninfected controls treated with PBS (*n* = 360 flies of each sex).

To study the effect of *Wolbachia* on Jak/Stat and Jnk signaling pathway activity in *D. melanogaster* flies infected with Zika virus, we assessed through qPCR the expression level of four representative readout genes. For the JAK/STAT pathway, there were no significant differences in *TotA* and *TotM* gene expression levels between Zika virus infected female flies carrying *Wolbachia* and those lacking the endosymbionts (Figure 6A). A similar gene expression pattern was also observed for *TotA* in male flies (Figure 6B). However, we found that male wild type flies containing *Wolbachia* had significantly higher *TotM* expression compared to those without the endosymbionts (Figure 6B). For Jnk signaling, there was significant upregulation of *Puckered*, but not *Basket*, in Zika virus infected wild type female adult flies carrying *Wolbachia* compared to those without the bacteria (Figure 6C). Finally, there were no statistically significant differences in *Puckered* and *Basket* gene expression levels between Zika virus infected male flies irrespective of their *Wolbachia* status (Figure 6D). These findings denote that *Wolbachia* endosymbionts can alter Jak/Stat and Jnk signaling activity in male and female *D. melanogaster* adults, respectively, during Zika virus infection.

## Discussion

Here we explored the potential effect of *Wolbachia* on the regulation of innate immune signaling and function of the *D. melanogaster* model against infection with the nonnatural pathogen, Zika virus. Our results provide evidence that *Wolbachia* can modulate the fly immune response in a sex-specific manner. More precisely, we show that the presence of *Wolbachia* prolongs the lifespan of adult flies, increases the survival of *D. melanogaster* females in response to Zika virus infection, and confers significant resistance to female, but not male, adult flies. Also, we find upregulation of the antimicrobial peptide gene *Drosocin* in Zika virus infected flies carrying the endosymbionts, but no changes in RNAi and Toll pathway regulated genes. Finally, we demonstrate that male and female flies containing *Wolbachia* have altered Jak/Stat and Jnk signaling activity during Zika virus infection. This information is important because it reveals that certain bacterial endosymbionts can be an important component of the host antiviral innate immune response against Zika virus and maybe other flaviviruses.

First, we observed that *Wolbachia* extends the lifespan of *D. melanogaster* Canton-S adult flies, which is conserved in both males and females. This information suggests that *Wolbachia* endosymbionts confer a fitness advantage to this *D. melanogaster* line. Previous research indicates that in most cases *Wolbachia* can provide fitness benefits for the insect host; however, the effect of *Wolbachia* on lifespan is variable and appears to depend on the genetic background of the fly and the strain of the endosymbiont (reviewed in Maistrenko et al., 2016). For instance, both increased and decreased lifespan effects have been found before in *D. melanogaster* and flies carrying the *wMel* strain of *Wolbachia* have shorter lifespan compared to flies free of endosymbionts (Min and Benzer, 1997; Martinez et al., 2015). In contrast to these previous findings, our results show that *wMel*-containing Canton-S flies have longer lifespan, which reinforce the concept that *D. melanogaster* longevity relies on the fly and *Wolbachia* strain.

The differences in *D. melanogaster* survival ability between female and male flies carrying or lacking *Wolbachia* following infection with Zika virus adds to our previous observations indicating sex-specific antiviral immune responses. In particular, we have recently shown that Zika virus infected *Dicer-2* female mutant flies have reduced food consumption rates compared to male mutants and Zika virus replicates at higher rates in adult *brat^chs^* mutants to cause motor dysfunction in a sex-dependent manner (Tafesh-Edwards et al., 2022, 2023). Also, *D. melanogaster* prophenoloxidase 1 (PPO1) is essential for male survival following Zika virus infection, while mutation of PPO2 triggers higher RNAi, Toll, Imd, and Jak/Stat immune signaling in female flies but not in male individuals, thus implying sex-specific immune responses during Zika virus infection (Tafesh-Edwards and Eleftherianos, 2023). Our current findings emphasize the complexity of antiviral defense in *D. melanogaster* through the potential involvement of *Wolbachia* endosymbionts in regulating host survival phenotypes and immune signaling activity differently in the two sexes.

In terms of the involvement of *Wolbachia* in altering the immune signaling in *D. melanogaster* challenged with Zika virus, we find that the presence of the endosymbionts in female or male adult flies fails to modify the expression of *Dicer-2* and *Ago-2* genes upon Zika virus infection. In *D. melanogaster*, RNAi plays an instrumental role in antiviral response (Carthew and Sontheimer, 2009). We paid attention to the expression of *Dicer-2* and *Ago-2* genes, which are components of the exogenous siRNA pathway that leads to the degradation of viral dsRNA molecules (Kim et al., 2006; Peters and Meister, 2007). More precisely, Dicer-2 recognizes exogenous dsRNAs and processes them into small-interfering RNAs (siRNAs), while Ago-2 is the central catalytic component of the RNA-induced silencing complex (RISC) and essential for antiviral defense (Galiana-Arnoux et al., 2006; van Rij et al., 2006). Previous research has indicated that *Wolbachia*-mediated protection of *D. melanogaster* adults against *Drosophila* C virus (DCV) is not RNAi dependent because fly mortality was slower in *Wolbachia* containing RNAi loss-of-function mutants compared to the *Wolbachia*-free individuals (Hedges et al., 2012). Our findings further imply the lack of participation of *Wolbachia* in altering RNAi signaling activity in both female and male flies following Zika virus injection.

We also find that with the exception of *Drosocin* transcriptional expression in female flies, *Wolbachia* presence in Zika virus infected *D. melanogaster* adults does not affect the mRNA levels of certain antimicrobial peptide-encoding genes. These results denote that Imd signaling activity can be modified in *Wolbachia*-containing female flies during Zika virus infection at least to some extent. The Imd pathway has been previously found to participate in the immune response of *D. melanogaster* against Cricket Paralysis virus, which is a natural viral pathogen of the fruit fly (Costa et al., 2009). In the context of Zika virus infection, the Imd pathway target gene *Diptericin A* has been shown to be upregulated in whole flies and heads, supporting the involvement of the Imd arm of the Rel/NF-κB pathway (Liu et al., 2018). Also, we have recently showed that Zika virus infection fails to activate Imd-mediated immunity in male *D. melanogaster* adult flies (Tafesh-Edwards and Eleftherianos, 2023). Interestingly, the *Drosocin* gene encodes two separate antimicrobial peptides with different specificity against distinct pathogens (Hanson et al., 2022). In addition, *Drosocin* was one of the antimicrobial peptide genes which was substantially upregulated in response to Sigma virus infection in *D. melanogaster* adult flies (Tsai et al., 2008). Whether the upregulation of *Drosocin* in female flies is directly attributed to *Wolbachia* or is an indirect effect as well as the role of this antimicrobial peptide in *Wolbachia* mediated protection to Zika virus infection will be the subject of future investigations.

Here we find that expression of the Jnk regulated gene *Puckered* increases during Zika virus infection in *Wolbachia* containing female flies. Previously, it has been shown that expression of the Puckered phosphatase, an inhibitor of Jnk activity, suppresses the expression of antimicrobial peptide genes in *D. melanogaster* (Delaney et al., 2006). Therefore, we speculate that the presence of *Wolbachia* specifically in female flies infected with Zika virus could possibly lead to the differential activation of Jnk signaling through the expression of *Puckered*, which in turn could potentially affect the expression of certain antimicrobial peptides, like Drosocin. The interaction between Jnk and Imd signaling by *Wolbachia* endosymbionts could be regulated through the TGF-beta activated kinase 1 (TAK1), which has been previously shown to act as an essential factor in Jnk signaling for the expression of antimicrobial peptides (Silverman et al., 2003; Delaney et al., 2006). Finally, we find that *Wolbachia*-containing male flies challenged with Zika virus have increased expression of the Jak/Stat regulated stress-induced *TotM* gene (Ekengren and Hultmark, 2001; Brun et al., 2006), and that male flies containing *Wolbachia* die at a slower rate by Zika virus compared to those lacking the endosymbiont, although there is no statistically significant difference. It is possible that *Wolbachia* presence in *D. melanogaster* males provides a slight protective effect against this virus and this effect is controlled through the activation of the Jak/Stat pathway. These various possibilities remain to be confirmed in future studies.

In conclusion, our findings point to a sex-specific effect of *Wolbachia* on the *D. melanogaster* immune response against Zika virus infection. The phenotypic effect of the endosymbionts is primarily demonstrated by the expansion in the survival of female adult flies after intrathoracic injection of Zika virus. The extended survival in *D. melanogaster* females is accompanied by reduced viral titers and changes in innate immune signaling. The latter is mainly expressed through the increased expression of the antimicrobial peptide gene *Drosocin* which is regulated by the Imd pathway, and the increased expression of the gene *Puckered* which is regulated by the Jnk pathway. The exact mechanism of decreased Zika virus replication in female flies carrying *Wolbachia* is currently unknown and it will be explored more in future studies. Further efforts will focus on testing whether the observed effects are specific to the *D. melanogaster* line Canton-S and the *Wolbachia pipientis* strain *w*Mel, and also to other *Drosophila* species. Considering that recent research has indicated that antiviral immunity in the fly is age dependent (Sheffield et al., 2021), it is intriguing to investigate the influence of age on the *Wolbachia* protective effect against Zika virus infection in female flies. Because hemocytes, autophagy, and the prophenoloxidase system contribute to *D. melanogaster* antiviral immunity (Lamiable et al., 2016; Tafesh-Edwards and Eleftherianos, 2023), potential input of *Wolbachia* to these aspects of the immune response against Zika virus will also be examined. Analyzing the dose-dependent and tissue-specific interaction of the innate immune response to oral infection with Zika virus in adult flies or larvae containing various concentration of the endosymbiotic bacteria will provide more detailed information about the prevalence of the current observations. Such information will contribute to a better understanding of the molecular and functional bases of endosymbiont-mediated resistance to Zika virus in mosquito vectors, which could reduce the transmission capacity of this viral pathogen and possibly other arboviruses.

## Data availability statement

The datasets presented in this study can be found in the NCBI Gene database. The accession numbers can be found in Table 1. Further inquiries can be directed to the corresponding author.

## Ethics statement

The manuscript presents research on animals that do not require ethical approval for their study.

## Author contributions

GT-E: Writing – review & editing, Visualization, Validation, Supervision, Methodology, Investigation, Formal analysis, Data curation, Conceptualization. MK: Writing – original draft, Visualization, Validation, Methodology, Investigation, Formal analysis. KM: Writing – review & editing, Methodology, Investigation. DB: Writing – review & editing, Validation, Resources, Methodology. SC: Writing – review & editing, Validation, Supervision, Resources, Conceptualization. IE: Writing – review & editing, Writing – original draft, Supervision, Project administration, Funding acquisition, Conceptualization.
